# No more counting sheep? Interoceptive not-distracting as a novel predictor of insomnia symptom severity in college students

**DOI:** 10.1093/sleepadvances/zpag062

**Published:** 2026-06-16

**Authors:** Lara R LoBrutto, Natalie D Dautovich, Patricia A Kinser, Bruce Rybarczyk, Joseph Dzierzewski

**Affiliations:** Department of Psychology, Virginia Commonwealth University, Richmond, VA, United States; Department of Psychology, Virginia Commonwealth University, Richmond, VA, United States; School of Nursing, Virginia Commonwealth University, Richmond, VA, United States; Department of Psychology, Virginia Commonwealth University, Richmond, VA, United States; National Sleep Foundation, Washington, DC, United States

**Keywords:** interoception, interoceptive awareness, insomnia symptoms, sleep disturbance, pre-sleep arousal

## Abstract

**Study Objectives:**

Emerging adults demonstrate vulnerability to pre-sleep arousal due to pervasive electronic device use, heightened rumination, and higher overall anxiety. To address this concern, this study sought to assess interoceptive awareness, a non-judgmental and trusting attitude toward body sensations that may reduce pre-sleep arousal, as a protective factor against insomnia symptoms during young adulthood.

**Methods and Measures:**

We assessed the association between interoceptive awareness, pre-sleep arousal, and insomnia symptom severity using cross-sectional data from university students (*N* = 420) in the mid-Atlantic United States. Participants completed the Multidimensional Assessment of Interoceptive Awareness-2, Pre-sleep Arousal Scale, and the Insomnia Severity Index. Data were analyzed using hierarchical linear and logistic regressions and a mediation analysis.

**Results:**

The “not-distracting” factor emerged as the strongest interoceptive predictor of insomnia symptoms in emerging adults. When adjusting for mood and demographic covariates, not-distracting significantly predicted pre-sleep arousal (b* = −0.12, *p* < .001). In the mediation model, pre-sleep arousal (95% CI: −0.25, −0.11) mediated the association between not-distracting and insomnia symptoms. Not engaging in distraction from pain and discomfort was associated with lower insomnia symptom severity via lower levels of cognitive and physiological arousal before sleep.

**Conclusions:**

Interoceptive not-distracting and pre-sleep arousal may be important factors to consider in the treatment of sleep disturbance in this age group. Findings suggest that specific training in interoceptive capacities could be a valuable addition to preventive interventions for sleep health in young adults.

**Statement of Significance**

Insomnia symptoms, which often involve cognitive and physiological arousal in the pre-sleep period, are common among young adults and may develop to clinically significant levels over the lifespan. This study is a novel assessment of interoceptive awareness in emerging adults as it relates to indicators of insomnia. Psychoeducation and training in interoceptive awareness, and particularly not-distracting, may be a valuable component of secondary interventions that limit the progression of insomnia.

## Graphical Abstract

**Graphical Abstract:**
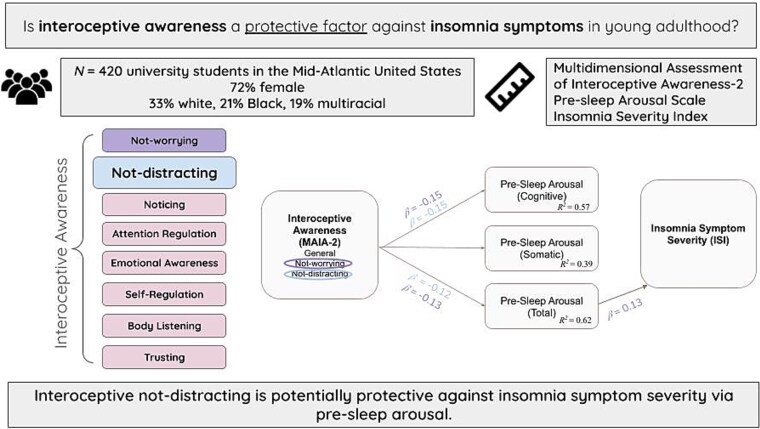

## Introduction

Insomnia, one of the most commonly occurring sleep–wake disorders, is a significant public health problem contributing to poor physical and mental health outcomes that reached an unprecedented global prevalence of 25% during the height of the COVID-19 pandemic [1, 2]. College students are particularly at risk of insomnia symptom development due to environmental influences, substance use, study habits, and mental health comorbidities [3–5]. Cognitive and physiological arousal before sleep contributes to insomnia symptoms in emerging adults [6]. The present study examined the hypothesized associations between interoceptive awareness and both pre-sleep arousal and insomnia symptoms and investigated pre-sleep arousal as a mediator of the interoceptive awareness and insomnia association among college students.

### Pre-sleep arousal

Pre-sleep arousal is a critical factor in the development and maintenance of insomnia. Arousal may be cognitive, resulting in mental activation via processes like rumination or not being able to shut off your thoughts, or physiological, characterized by autonomic nervous system activation, with reactions such as increased heart rate [7]. Often, there is interplay between the two [8]: for example, an individual might notice that their heart is racing, followed by a mental preoccupation with their heart rate, leading to preoccupying thoughts (e.g. “I’m never going to be able to sleep in this state”) and behaviors (e.g. tracking one’s pulse or monitoring heart rate on a wearable device). Numerous studies demonstrate that nighttime cognitive arousal is higher in individuals with insomnia than in good sleepers [9]. Cognitive arousal such as worrying and monitoring are associated with longer sleep onset latency, worse sleep quality, and shorter total sleep time [10, 11]. Although most research has focused on cognitive arousal, physiological arousal is also linked to higher sleep latency, and lower total sleep time, and is observed at higher rates in individuals with insomnia compared with good sleepers [8, 11, 12]. In college undergraduates, pre-sleep arousal is a predictor of insomnia development [6]. Binge-watching television and nighttime cell phone use, both of which are common in this age group, increase pre-sleep cognitive activity in a way that increases the likelihood of sleep disturbance [13, 14]. Additionally, emerging adults are at greater risk of anxiety and depression, which can contribute to pre-sleep arousal through cognitive processes (e.g. worry and rumination) and somatic symptoms [15–17].

Pre-sleep arousal, cognitive, and physiological states that are incompatible with sleep onset has the potential to be reduced via training of interoceptive awareness. Interoceptive awareness, described by some as interoceptive sensibility and adaptive body awareness, involves a healthy cognitive conception of body sensation comprised of non-judgmental and trusting attention [18, 19]. This awareness includes noticing uncomfortable internal signals (e.g. tense muscles) when they arise but not letting this noticing become preoccupation. At the same time, it involves finding solace in the body when external stressors create destabilization (e.g. focusing on the breath to reduce anxiety at bedtime) [20].

### Interoception and sleep

Interoception, although understudied in a young adult population, may be an important protective factor against the progression of insomnia among college students. A small body of research has investigated interoceptive awareness in relation to sleep and has shown that in individuals with insomnia, some components of interoceptive awareness may be maladaptively heightened. For example, although individuals with poor sleep and insomnia demonstrate increased somatic awareness and interoceptive attention [8, 21], they are more likely to identify painful or uncomfortable sensations compared to positive and pleasant ones [22]. Additionally, individuals with insomnia disorder have different interoceptive profiles from good sleepers, with heightened averages on some facets and suppressed scores on others [23]. There is minimal direct evidence of a positive association between interoceptive awareness and healthy sleep; however, several components of interoceptive awareness are associated with better overall self-reported sleep quality in young adults, suggesting it may play a role in reduced sleep disturbance [24]. Overall, there is a need to better understand associations between interoceptive awareness factors, pre-sleep arousal, and insomnia symptom severity.

College students are at risk for heightened pre-sleep arousal due to behavioral patterns [13, 14] and greater prevalence of anxiety and depression in this age cohort [25]. Interoceptive awareness is posited as a protective factor against the development and maintenance of sleep disturbance via reduced pre-sleep arousal. The overall objective of the study was to evaluate whether interoceptive awareness is a factor in pre-sleep arousal and insomnia symptoms in undergraduate students. Specifically, we examined the associations between three factors of interoceptive awareness (general, not-worrying, and not-distracting), pre-sleep arousal, and insomnia symptoms in college students. We aimed to (a) investigate the association between interoceptive awareness and pre-sleep arousal, (b) evaluate interoceptive awareness as a predictor of insomnia symptom severity, and (c) explore pre-sleep arousal as a mediator of the association between interoceptive awareness and insomnia. We hypothesized that interoceptive awareness would be negatively associated with pre-sleep arousal and insomnia symptom severity, and pre-sleep arousal would mediate the interoceptive awareness and insomnia symptom severity association.

## Materials and methods

### Participants and procedure

The current study reports on a subset of data collected from a parent study with undergraduate students attending a large public, urban university in the mid-Atlantic United States in 2023. Recruitment occurred via research requirement for course credit and course extra credit. No monetary compensation was provided and students had the option to engage in alternative activities for research or extra credit. Student participants were required to be age 18 or older and able to complete study materials written in English. Participants completed an online survey via an electronic data capture system (REDCap), ~45 min in length, that included demographic questions, sleep questionnaires, and a range of psychosocial measures. The current study used a subset of these measures. Attention checks were interspersed throughout the survey to increase validity of the data. The study methods were approved by the Institutional Review Board.

### Measures

#### Demographic factors

The baseline questionnaire asked participants to report current age, gender identity, racial and ethnic identity, and socioeconomic status.

#### Mood

Mood was measured using the Generalized Anxiety Disorder-7 (GAD-7) [26] and the Patient Health Questionnaire (PHQ-9) [27]. The GAD-7 is composed of 7 items in which participants report how bothersome a stated problem has been in the last 2 weeks. Likert style responses range from 0 (*not at all*) to 3 (*nearly every day*). Total scores range from 0 (*no anxiety*) to 21 (*severe anxiety*). The PHQ-9 consists of 9 items in which participants report how bothersome a stated problem has been in the last 2 weeks. Likert style responses range from 0 (*not at all*) to 3 (*nearly every day*). Total scores range from 0 (*no depression*) to 27 (*severe depression*). Because the PHQ-9 references sleep in one item (#3, “Trouble falling or staying asleep, or sleeping too much”), this item was removed the total score prior to analyses.

#### Pre-sleep arousal

The Pre-Sleep Arousal Scale is a validated self-report measure for assessing an individual’s state prior to sleep onset [7]. It consists of 16 items with answer choices ranging from 1 (*not at all*) to 5 (*extremely*). Half of the items refer to cognitive arousal, such as “Can’t shut off your thoughts,” and the other half refer to somatic or physiological arousal, including “A tight, tense feeling in your muscles.” Total scores can range from 16 to 80, with higher scores indicating greater levels of arousal. The cognitive and physiological subscales, as well as the total score, were utilized in analyses.

#### Sleep quality

The Pittsburgh Sleep Quality Index (PSQI) assesses self-reported sleep quality over the previous one-month interval and was included to better describe sleep characteristics of the present sample. Responses are made on a 4-point Likert scale with the exception of a few write-in answers. The PSQI includes 19 items and produces seven subscales, including sleep quality, sleep latency, sleep duration, habitual sleep efficiency, sleep disturbances, use of sleep medication, and daytime dysfunction. Sleep duration is defined as total time in bed minus time spent awake. Sleep onset latency is defined as the amount of delay between the attempt to initiate sleep and actual sleep onset and is calculated based on responses to two items. Sleep efficiency refers to the amount of sleep relative to the time in bed and is calculated based on the responses to three items. The seven component scores are used to calculate a global score ranging from 0 to 21, and a score of less than five indicates good sleep, while greater than five indicates poor sleep. The scale has been validated in a college sample, with high internal consistency (α = 0.87) [28].

#### Insomnia severity

The Insomnia Severity Index is a validated measure used to assess insomnia symptoms [29]. The scale contains seven items with Likert-style responses ranging from 0 to 4. Scores are summed to produce a total score between 0 and 28. Scores of >10 are indicative of clinically significant insomnia [29].

#### Interoceptive awareness

The Multidimensional Assessment of Interoceptive Awareness Version 2 (MAIA-2) was used to assess interoceptive awareness. It consists of 37 items with answer choices ranging from 0 (*never*) to 5 (*always*). Higher subscale scores indicate more IA. It contains eight subscales. The subscales are intended to be considered separately rather than to be used to derive a total score; however, a General MAIA factor consisting of six of the eight subscales (Noticing, Attention Regulation, Emotional Awareness, Self-Regulation, Body Listening, and Trusting) has been validated [30]. The General factor captures “perception of body changes and rhythms” [30]. Furthermore, two of the subscales, Not-worrying and Not-distracting, show distinct and valid construct validity [30, 31]. Not-worrying and Not-distracting capture disengagement from catastrophizing and avoidance, respectively, relating to physical discomfort or pain [20]. We used the General MAIA factor (scores range from 0 to 30), the Not-worrying subscale (scores range from 0 to 5), and the Not-distracting subscale (scores range from 0 to 5) as the three IA factors in our analyses.

### Analyses

A power analysis conducted using G*power software [32] showed that a sample of 209 participants provided 80% power to detect a small-to-medium effect size of *f*^2^ ≥ .075, with a type-I error rate of α = .05, using eight predictors. All analyses were completed using SPSS version 28. Prior to analyses, normality tests were applied to assess for normal distribution of data via tests of skewness and kurtosis (critical values ± 2) [33]. Additionally, data assumptions were reviewed to ensure that assumptions of independence, normality, multicollinearity, and homoscedasticity were met.

Gender identity, racial identity, socioeconomic status, and mood (i.e. anxiety and depression) were selected as potential covariates for all regression analyses given their significant associations with sleep outcomes [34–40]. For the purposes of the analyses, racial/ethnic and gender demographic groups comprising <10% of the total sample were grouped together. Additionally, individuals self-identifying as more than one race/ethnicity were coded into a new multiracial category. All demographic variables were dummy coded to ensure that analysis results were easily interpretable. Given the known associations between demographic and mood variables and sleep outcomes [40–42] and the less known associations between interoceptive awareness and sleep measures, interoceptive awareness was entered as the first step in all the below described models to better understand its unique association with sleep. Mood and demographic predictors were then entered into the models in steps 2 and 3, respectively. To examine aim A, three hierarchical regressions were conducted to predict cognitive, physiological, and overall pre-sleep arousal from interoceptive awareness (general factor, not-worrying, and not-distracting). To examine aim B, a hierarchical linear regression was conducted predicting insomnia symptom severity from interoceptive awareness (general factor, not-worrying, and not-distracting). To examine aim C, a mediation analysis was run to see if pre-sleep arousal mediated the relationship between IA subscales and insomnia symptom severity while controlling for gender, race/ethnicity, and mood variables. The PROCESS macro [43] with 5000 bootstrapped samples was used for this analysis.

## Results

The final sample consisted of 420 participants, after 159 records were removed due to not passing validity checks or implausible data or outliers. Participants had a mean age of 19.07 years (*SD* = 2.11). The majority of participants identified as female (72.4%) and middle class (65.6%). Although self-identified white participants were the largest group (33.2%), the sample was racially diverse with 20.7% Black/African American, 19% multiracial, 16.1% Asian American/Pacific Islander, and 10.4% Hispanic/Latinx participants. Participants slept an average of 7.1 h per night with average latency of 34.6 min and efficiency of 83.7%. More than half of the sample reported insomnia symptoms, with the majority falling into the mild range. Complete descriptive statistics are included in Table 1.

**Table 1:** Sample descriptive statistics

**Variable**	Descriptives
Age, years *M* (*SD*)	19.07 (2.11)
Gender identity *N* (%)^*^	
Female	305 (72.4)
Male	96 (22.8)
Gender minority	19 (4.5)
Race/ethnicity *N*(%)^†^	
White	144 (33.2)
Black/African American	90 (20.7)
Asian American/Pacific Islander	70 (16.1)
Hispanic/Latinx	45 (10.4)
Multiracial	80 (19.0)
Socioeconomic status *N* (%)^‡^	
Poor or low income	26 (6.2)
Working class	91 (21.6)
Middle class	276 (65.6)
Rich or upper class	27 (6.4)
Caffeine use *N* (%)^§^	343 (82.9)
Alcohol use *N* (%)^§^	101 (24.0)
Marijuana use *N* (%)^§^	72 (17.1)
Sleep medication use—past month *N* (%)^||^	
None	308 (73.2)
Less than once per week	46 (10.9)
Once or twice per week	37 (8.8)
3+ times per week	27 (6.4)
Sleep interruption due to pain *N* (%)^||^	
None	276 (66.8)
Less than once per week	74 (17.9)
Once or twice per week	47 (11.4)
3+ times per week	16 (3.9)
Interoceptive awareness *M* (*SD*)	
Not worrying	2.4 (0.8)
Not distracting	2.0 (1.0)
General	18.0 (4.6)
Pre-sleep arousal *M* (*SD*)	
Cognitive	23.3 (7.6)
Somatic	15.5 (6.5)
Total	38.8 (12.7)
PSQI *M* (*SD*)	(178.4, 87.9)
Latency (min)	34.6 (31.3)
Duration (h)	7.1 (1.3)
Efficiency (%)	83.7 (13.1)
Global Score	6.3 (3.1)
Insomnia severity *N* (%)	
Mild	176 (41.8)
Moderate	67 (15.9)
Severe	9 (2.1)
Depressive symptoms *M* (*SD*)	9.9 (6.2)
Minimal	99 (23.5)
Mild	111 (26.4)
Moderate	101 (24.0)
Moderately severe	63 (15.0)
Severe	32 (7.6)
Anxiety symptoms *M* (*SD*)	9.4 (5.6)
Minimal	93 (22.1)
Mild	141 (33.5)
Moderate	102 (24.2)
Severe	80 (19.0)

^*^Options available for describing gender identity included male, female, non-binary/third gender, transgender, agender, and genderfluid, with options to self-describe or not disclose.

^†^Multiple boxes could be selected for racial or ethnic identity, which included Asian, Black/African-American, White, Hispanic/Latinx, Native American, and Pacific Islander, with an option not to disclose.

^‡^Participants were asked to report perceived socioeconomic status from the following answer choices: poor or low-income, working class, middle class, and rich or upper-class.

§ Participants reported general current substance use as well as daily frequency.

|| These items were collected via the Pittsburgh Sleep Quality Index items 7 and 5i respectively.

### Associations between interoceptive awareness and pre-sleep arousal

Hierarchical linear regressions (Tables 2 and 3) were run to examine the association between general interoceptive awareness, not-worrying interoceptive awareness, and not-distracting interoceptive awareness and pre-sleep cognitive, somatic, and total arousal. In the cognitive arousal regression, block 1 resulted in interoceptive awareness as a significant predictor, *F*(3, 359) = 20.87, *p* < .001, *R*^2^ = 0.15, with not-distracting, b* = −0.35, *p* < .001, and not-worrying, b* = −0.15, *p* = .002, as individual significant predictors. In Block 2, the addition of mood variables significantly improved the model, Δ*R^2^* = 0.42, *p* < .001, and not-distracting, b* = −0.16, *p* < .001, anxiety, b* = 0.29, *p* < .001, and depression, b* = 0.46 and *p* < .001, were significant predictors. In block 3, adding gender and race did not significantly improve the model.

**Table 2:** Hierarchical regression results for pre-sleep arousal (cognitive and somatic)

Variable	PSAS cognitive				PSAS somatic			
	*B*	95% CI for *B*	SE *B*	β	*B*	95% CI for *B*	SE *B*	β
Step 1								
Gen	−0.13	[−0.29, 0.03]	0.08	−0.08	−0.13	[−0.28, 0.01]	0.07	−0.09
ND	−2.71^***^	[−3.46, −1.96]	0.38	−0.35^***^	−1.53^***^	[−2.20, −0.85]	0.34	−0.23^***^
NW	−1.48^**^	[−2.41, −0.56]	0.47	−0.15^**^	−0.59	[−1.41, 0.24]	0.42	−0.07
*R* ^2^ = 0.15^***^, $\Delta$*R*^2^ = 0.15^***^					*R* ^2^ = 0.07^***^, $\Delta$*R*^2^ = 0.07^***^			
Step 2								
Gen	0.04	[−0.07, 0.16]	0.06	0.03	0.01	[−0.11, 0.13]	0.06	0.01
ND	−1.23^***^	[−1.80, −0.66]	0.29	−0.16^***^	−0.41	[−0.98, 0.16]	0.29	−0.06
NW	−0.66	[−1.36, 0.04]	0.36	−0.08	−0.06	[−0.76, −0.65]	0.36	−0.01
Anx	0.42^***^	[0.27, 0.56]	0.07	0.29^***^	0.26^***^	[0.12, 0.41]	0.07	0.22^***^
Dep	0.57^***^	[0.42, 0.72]	0.08	0.46^***^	0.49^***^	[0.34, 0.64]	0.08	0.42^***^
*R* ^2^ = 0.57^***^, $\Delta$*R*^2^ = 0.41^***^					*R* ^2^ = 0.40^***^, $\Delta$*R*^2^ = 0.32^***^			
Step 3								
Gen	0.05	[−0.07, 0.17]	0.06	0.03	0.01	[−0.11, 0.14]	0.06	0.01
ND	−1.19^***^	[−1.70, −0.56]	0.29	−0.15^***^	−0.39	[−0.97, 0.19]	0.29	−0.06
NW	−0.68	[−1.45, −0.05]	0.36	−0.07	−0.06	[−0.74, 0.70]	0.37	−0.00
Anx	0.43^***^	[0.25, 0.53]	0.07	0.32^***^	0.26^**^	[0.11, 0.40]	0.08	0.22^**^
Dep	0.56^***^	[0.42, 0.67]	0.07	0.40^***^	0.49^***^	[0.34, 0.65]	0.08	0.41^***^
Gender	−0.69	[−2.11, 0.72]	0.72	−0.04	0.27	[−1.15, 1.68]	0.72	0.02
Race	−0.12	[−0.68, 0.45]	0.29	−0.02	0.24	[−0.32, 0.81]	0.29	0.04
*R* ^2^ = 0.57^***^, $\Delta$*R*^2^ = 0.00					*R* ^2^ = 0.40^***^, $\Delta$*R*^2^ = 0.00			

CI = confidence interval; LL = lower limit; UL = upper limit; Gen = MAIA general factor; ND = MAIA not-distracting factor; NW = MAIA not-worrying factor; Anx = anxiety; Dep = depression.

^*^ *p* < .05; ^**^p < .01; ^***^p < .001.

**Table 3:** Hierarchical regression results for pre-sleep arousal (total)

PSAS total							
Variable	*B*	95% CI for *B*		SE *B*	β	*R* ^2^	$\Delta$ *R* ^2^
		LL	UL				
Step 1						0.13^***^	0.13^***^
Gen	−0.26	−0.53	0.01	0.14	−0.10		
ND	−4.22^***^	−5.47	−2.97	0.64	−0.33^***^		
NW	−2.03^*^	−3.57	−0.49	0.78	−0.13^*^		
Step 2						0.61^***^	0.48^***^
General	0.06	−0.13	0.25	0.10	0.02		
ND	−1.59^***^	−2.48	−0.71	0.45	−0.12^***^		
NW	−0.64	−1.73	0.45	0.56	−0.04		
Anx	0.68^***^	0.46	0.91	0.11	0.31^***^		
Dep	1.07^***^	0.84	1.31	0.12	0.47^***^		
Step 3						0.61^***^	0.00
Gen	0.07	−0.12	0.25	0.10	0.02		
ND	−1.53^**^	−2.43	−0.63	0.46	−0.12^**^		
NW	−0.64	−1.75	0.48	0.57	−0.04		
Anx	0.68^***^	0.45	0.91	0.12	0.31^***^		
Dep	1.06^***^	0.82	1.29	0.12	0.46^***^		
Gender	−0.55	−0.79	1.69	1.14	−0.02		
Race	0.21	−0.69	1.11	0.46	0.02		

CI = confidence interval; LL = lower limit; UL = upper limit; Gen = MAIA general factor; ND = MAIA not-distracting factor; NW = MAIA not-worrying factor; Anx = anxiety; Dep = depression.

^*^ *p* < .05; ^**^p < .01; ^***^p < .001.

For pre-sleep somatic arousal, interoceptive awareness was a significant predictor of arousal when entered in the first block, *F*(3, 359) = 8.36, *p* < .001, *R*^2^ = 0.06, with only not-distracting, b* = −0.23, *p* < .001, being a significant predictor. In block 2, the addition of mood significantly improved the model, Δ*R*^2^ = 0.32, *p* < .001. Within this block, only depression, b* = 0.42, *p* < .001, and anxiety, b* = 0.22, *p* < .001, remained statistically significant. In block 3, adding gender and race did not significantly improve the model.

Interoceptive awareness was also a significant predictor of total pre-sleep arousal when entered in the first block, *F*(3, 357) = 18.09, *p* < .001, *R*^2^ = 0.13. Within the block, not-distracting, b* = −0.33, *p* < .001, and not-worrying, b* = −0.13, *p* = .01, were significant predictors. In block 2, the addition of mood significantly improved the model, Δ*R*^2^ = 0.47, *p* < .001. Within this block, not-distracting (b* = −0.12, *p* < .001), depression (b* = 0.47, *p* < .001), and anxiety (b* = 0.31, *p* < .001) were statistically significant. In block 3, adding gender and race did not significantly improve the model.

### Associations between interoceptive awareness and insomnia symptom severity

A hierarchical linear regression (Table 4) was run to examine the associations between general interoceptive awareness, not-worrying interoceptive awareness, and not-distracting interoceptive awareness and insomnia symptom severity. Interoceptive awareness was a significant predictor of insomnia symptom severity when entered in the first block, *F*(3, 351) = 8.20, *p* < .001, *R*^2^ = 0.06, with only not-distracting, b* = −0.23, *p* < .001, being a significant predictor. In block 2, the addition of mood significantly improved the model, Δ*R*^2^ = 0.35, *p* < .001. Within this block, only depression, b^*^ = 0.05, *p* < .001, and anxiety, b^*^ = 0.17, *p* = .01, remained statistically significant. In block 3, adding gender and race did not significantly improve the model.

**Table 4:** Hierarchical regression predicting insomnia symptom severity

ISI							
Variable	*B*	95% CI for *B*		SE *B*	β	*R* ^2^	$\Delta$ *R* ^2^
		LL	UL				
Step 1						0.06^***^	0.06^***^
Gen	−0.09	−0.21	0.03	0.06	−0.08		
ND	−1.28^***^	−1.84	−0.73	0.28	−0.23^***^		
NW	−0.37	−1.05	0.31	0.35	−0.06		
Step 2						0.41^***^	0.35^***^
General	0.02	−0.08	0.12	0.05	0.02		
ND	−0.34	−0.80	0.12	0.24	−0.06		
NW	−0.03	−0.60	0.54	0.29	−0.00		
Anx	0.16^**^	0.04	0.28	0.06	0.17^**^		
Dep	0.42^***^	0.32	0.53	0.49	0.05^***^		
Step 3						0.42^***^	0.00
Gen	0.07	−0.12	0.25	0.10	0.02		
ND	−1.53	−2.43	−0.63	0.46	−0.12		
NW	−0.64	−1.75	0.48	0.57	−0.04		
Anx	0.68^***^	0.45	0.91	0.12	0.31^***^		
Dep	1.06^***^	0.82	1.29	0.12	0.46^***^		
Gender	−0.55	−1.74	0.65	0.61	−0.05		
Race	0.28	−0.19	0.76	0.24	0.06		

CI = confidence interval; LL = lower limit; UL = upper limit; Gen = MAIA general factor; ND = MAIA not-distracting factor; NW = MAIA not-worrying factor; Anx = anxiety; Dep = depression.

^*^ *p* < .05; ^**^p < .01; ^***^p < .001.

### Pre-sleep arousal as a mediator of interoceptive not-distracting and insomnia symptom severity

**Figure 1:**
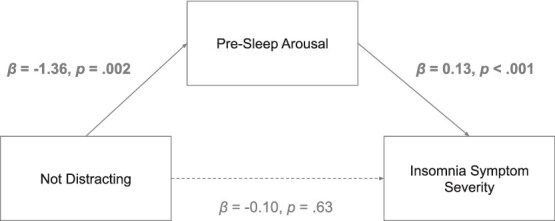
Mediation model of interoceptive not-distracting and insomnia symptom severity.

No indirect effect was found for MAIA General (95% CI: −0.02, 0.03) or MAIA Not Worrying (95% CI: −0.04, 0.00) linking total pre-sleep arousal and insomnia symptom severity. However, the mediation model containing MAIA not-distracting was significant, *R*^2^ = 0.62, *F*(3, 373) = 202.45, *p* < .001 (Figure 1). Total pre-sleep arousal (95% CI: −0.32, −0.06) mediated the relationship between MAIA not-distracting and insomnia symptom severity when controlling for gender, race, anxiety, and depression.

## Discussion

The present study found that interoceptive awareness is linked to insomnia symptom severity in college students, with pre-sleep arousal playing a meaningful role. Across various models, general interoceptive awareness, not-worrying, and not-distracting demonstrated differing associations with pre-sleep arousal and insomnia symptom severity. Not-distracting, characterized by not ignoring or tuning out uncomfortable body sensation, emerged as the most reliable factor related to both pre-sleep arousal and insomnia symptom severity in this population. Participants not engaging in distraction had lower self-reported levels of cognitive and overall wakefulness before sleep. Based on this finding, young adults who are higher in not-distracting are less likely to avoid distressing thoughts or occupy themselves in order to bypass unpleasant sensation.

Although distraction (e.g. counting sheep) is a frequently recommended pop technique for falling asleep, this is among several studies demonstrating that distraction is an ineffective strategy for improving sleep outcomes among good sleepers and individuals with insomnia [9]. It is possible that mentally conjuring a distraction actually increases cognitive activity rather than reducing it in a way that prepares the mind and body for sleep. In contemporary life, distraction often takes the form of smartphone use prior to bed, which has been associated with poor sleep and mental health in young adults [44, 45]. This finding also may be explained by individuals less likely to engage in distraction processing difficult emotional content during the daytime, with the result being lower levels of sleep interference.

Although the direct association between interoceptive not-distracting and insomnia symptom severity was attenuated with the addition of depression and anxiety symptoms, the indirect association with pre-sleep arousal held. These results suggest that not-distracting is potentially indirectly linked to insomnia symptom severity and one bidirectional pathway may be through mood. Interoceptive awareness and behavioral health are closely related [46], which may explain this finding. For example, greater levels of depressive symptoms co-occur with less noticing and trusting of body sensation and regulation of the corresponding emotion [47–50], and trait anxiety is linked to negative evaluation of body signals [51].

It is also possible that what appeared to be insomnia symptoms could be otherwise characterized due to the unique attributes of emerging adulthood. Behaviorally induced insufficient sleep syndrome (i.e. self-imposed sleep limitations and daytime sleepiness) is experienced by 1 in 10 college students [52]. Consequently, some insomnia symptoms reported in the present study could be representative of behaviorally-induced sleep limitations that may less related to cognitive and physiological, and thus interoceptive, processes.

As hypothesized, promoting adaptive interoceptive awareness in the form of not-distracting may have potential to counteract pre-sleep arousal during emerging adulthood. Early detection and treatment of insomnia in young adults is important for preventing continuation and mitigating downstream behavioral, psychological, and academic consequences [53–56]. A practice of not-distracting could involve bringing attention to the present moment, as one might by doing a body scan (i.e. attending to each part of the body, beginning from the toes, and moving upward). Beyond tools for individual use, therapeutic interventions that allow practice of interoceptive awareness, such as Mindful Awareness in Body Oriented Therapy, have been successfully used in the context of substance use disorders and may be adapted to address sleep disturbance [57]. Additionally, Acceptance and Commitment Therapy (ACT) is a therapeutic approach that teaches present moment awareness and has demonstrated efficacy for the treatment of insomnia in adults [58–60]; an ACT-based approach with targeted interoceptive awareness training could be especially valuable for college-age individuals facing an abundance of distractions. Future studies could explore the addition of interoceptive skill-building to secondary prevention interventions for insomnia with the goal of slowing or preventing the transition from acute to chronic manifestations in young adults.

This study has several strengths and limitations that need to be acknowledged. Notable strengths include the relatively large and diverse sample of students in terms of race/ethnicity and socioeconomic status, use of validated measures, novel conceptualization of interoceptive awareness [30], and first investigation of interoceptive awareness and pre-sleep arousal in this population. Notable limitations include not incorporating objective or gold standard assessments of interoception (e.g. heart rate variability) [61, 62] or insomnia (e.g. clinical interview and sleep diaries), and the cross-sectional nature of the study which prevented causal inferences.

## Conclusion

The current study provided evidence of an association between interoceptive awareness, specifically not-distracting, and pre-sleep arousal in college students, as well as demonstrating that pre-sleep arousal may underlie the association between not-distracting and insomnia. These findings contribute to a small but growing body of literature on interoceptive awareness and provide novel links between interoceptive awareness and sleep outcomes in this age group. Additionally, these findings have notable implications, with the potential to inform future interventions aimed at limiting the progression from acute to chronic insomnia in young adults. Future research may assess similar associations with the incorporation of daily and objective sleep measures.

## Data Availability

Data underlying this article will be shared on reasonable request to the corresponding author.
